# SERVICE PROVISION IN RESIDENTIAL CARE COMMUNITIES FOR RESIDENTS WITH DEMENTIA AND DEPRESSION

**DOI:** 10.1093/geroni/igz038.1869

**Published:** 2019-11-08

**Authors:** Manisha Sengupta, Christine Caffrey

**Affiliations:** 1 CDC/National Center for Health Statistics, Hyattsville, Maryland, United States; 2 Centers for Disease Control and Prevention/National Center for Health Statistics, Hyattsville, Maryland, United States

## Abstract

Assisted living and similar residential care communities (RCCs) are an important source of care for older adults, many of whom have dementia or depression. In 2016, 42% of residents in RCCs were diagnosed with dementia and 31% were diagnosed with depression. About two-thirds of RCCs (63%) provided social work and mental health services and 37% provided neither service. Using the 2016 National Study of Long-Term Care Providers, this study includes bivariate and ANOVA modeling to (1.) examine the variation in dementia and depression between RCCs that provide social work and mental health services and those that do not, and (2.) assess if there is an interaction between provision of services and RCC bed size in their association with dementia and depression. Bivariate results show that the prevalence of dementia does not vary by service provision, but the prevalence of depression does. The percent of residents with depression varied from 34% in RCCs that provided both social work and mental health services to 31% in RCCs that provided only one service to 28% in RCCs that provided neither service. The prevalence of dementia was around 4 in 10 residents, regardless of service provision. Fitting a two-way ANOVA model for dementia indicated that the effect was 4 percentage points higher for RCCs that provided services than those that did not, but only among small RCCs. Findings from this study can inform strategies to care for the needs of residents with dementia and depression across the various sizes of RCCs.

